# Genome-Wide Identification of the *JAZ* Gene Family in Garlic (*Allium sativum* L.) and the Functional Role of *AsJAZ17* in Salt Tolerance

**DOI:** 10.3390/plants15101543

**Published:** 2026-05-19

**Authors:** Zhenyu Cao, Na Li

**Affiliations:** College of Horticulture and Plant Protection, Inner Mongolia Agricultural University, Hohhot 010018, China; lsf15048456237@163.com

**Keywords:** *Allium sativum*, *JAZ* gene family, salt tolerance, jasmonate signaling

## Abstract

Jasmonate ZIM-domain (JAZ) proteins are pivotal repressors in the jasmonate (JA) signaling pathway, yet their specific roles in garlic (*Allium sativum*) remain largely unexplored. In this study, 28 *AsJAZ* genes were identified through a genome-wide analysis. The expansion of this family was primarily driven by whole-genome duplication events, with a significant majority (71.43%) of members belonging to a lineage-specific clade, Subfamily E. While AsJAZ proteins harbor conserved TIFY and Jas domains, they exhibit diverse gene structures and subcellular localization patterns. Notably, AsJAZ17 is strictly localized to the nucleus, whereas AsJAZ16 shows a nucleocytoplasmic distribution, suggesting potential functional compartmentalization within the family. Transcriptomic and qRT–PCR analyses revealed that most *AsJAZ* genes are responsive to heat, salt, and methyl jasmonate (MeJA) treatments. Protein–protein interaction (PPI) modeling and yeast two-hybrid (Y2H) assays confirmed that AsJAZ17 physically interacts with the MYC2 transcription factor, identifying it as a key regulator within the conserved COI1-JAZ-MYC2 signaling module. Functional validation demonstrated that overexpression of *AsJAZ17* in Arabidopsis significantly enhances salt tolerance. This improvement is attributed to an optimized growth-defense trade-off and a reinforced antioxidant defense system, as evidenced by the increased activities of superoxide dismutase (SOD), peroxidase (POD), and catalase (CAT), which collectively maintain reactive oxygen species (ROS) homeostasis under stress. These findings provide comprehensive insights into the evolutionary and functional landscape of the garlic *JAZ* family and identify *AsJAZ17* as a promising candidate gene for molecular breeding to improve abiotic stress resilience in Allium crops.

## 1. Introduction

Jasmonic acid (JA) and its bioactive derivative, jasmonoyl-isoleucine (JA-Ile) [1] are essential lipid-derived phytohormones that orchestrate the delicate balance between plant growth, development, and environmental adaptation. In the absence of stress, JA signaling is maintained in a repressed state by Jasmonate ZIM-domain (JAZ) proteins, which act as transcriptional repressors by sequestering key transcription factors (TFs), such as MYC2, and recruiting co-repressors like NINJA [2]. Upon perception of external stimuli, the rapid accumulation of intracellular JA-Ile levels facilitates the interaction between JAZ proteins and the SCF-COI1 ubiquitin ligase complex, where JA-Ile acts as a “molecular glue”. This interaction triggers the degradation of JAZ proteins via the 26S proteasome pathway, thereby releasing the sequestered TFs to activate large-scale adaptive physiological programs [3,4]. Although this signaling cascade can be further fine-tuned by diverse post-translational modifications, the functional versatility of the JA response is primarily driven by the structural conservation and evolutionary diversification of the JAZ family itself [5].

Functioning as the central repressive hubs of the JA pathway, prototypical JAZ proteins are characterized by two highly conserved domains: the N-terminal ZIM domain, which mediates protein dimerization and co-repressor recruitment, and the C-terminal Jas domain, essential for JA-Ile-dependent JAZ-COI1 interaction and degradation [6]. From an evolutionary perspective, the *JAZ* gene family has undergone significant expansion in higher plants, particularly in angiosperms, primarily driven by whole-genome duplication (WGD) and tandem duplication events [7]. While the model plant *Arabidopsis thaliana* possesses 12 JAZ members, the family size is markedly larger in polyploid crops, which facilitates functional divergence, or neofunctionalization, enabling plants to adapt to increasingly complex ecological niches. Recent advances have further highlighted that JAZ proteins function as signaling hubs, integrating JA signaling with other hormonal pathways to mediate responses to abiotic stresses. For instance, JAZ members have been identified as key regulators of drought and cold tolerance in Arabidopsis and tomato by modulating reactive oxygen species (ROS) scavenging and repressing stress-responsive activators [8,9]. Furthermore, JAZ proteins integrate epigenetic regulation and nutrient sensing, as evidenced by the OsJAZ8-HDA19 module in rice salt tolerance and the PHR1-JAZ-MYC2 module in phosphate starvation responses [10,11].

Despite the critical roles identified for JAZ proteins in model species and major cereal crops, their regulatory potential remains largely uncharacterized in many globally significant vegetable crops, such as garlic (*Allium sativum* L.). Garlic is widely recognized for its abundance of bioactive constituents, including allicin, polyphenolic compounds, and fructans, which collectively contribute to its pronounced antioxidant, antimicrobial, and health-enhancing properties. The garlic genome is notably large, with an estimated size of approximately 16 Gb and comprising about 91.3% repetitive sequences. Phylogenomic evidence indicates that the garlic genome has undergone three distinct WGD events: the first two occurring prior to the divergence from *Asparagus officinalis* approximately 80.8 million years ago (Mya), and a third event inferred to have occurred around 17.9 Mya [12], followed by a recent transposable element burst approximately 0.2–0.3 Mya [13]. These profound genomic events not only contributed to the massive expansion of the garlic genome but also provided the genetic basis for the duplication, diversification, and neofunctionalization of many gene families, including the JAZs.

In the present study, we performed a comprehensive genome-wide identification of the *JAZ* gene family in *A. sativum* to elucidate its evolutionary trajectory and potential roles in stress adaptation. We characterized the chromosomal distribution, gene structures, and conserved motifs of the identified *AsJAZ* members, and further investigated their phylogenetic relationships with JAZs from other representative species. To gain insights into their functional roles, we analyzed the expression profiles of *AsJAZ* genes under various abiotic stresses and during different developmental stages. This study provides a foundation for understanding the JA-mediated regulatory networks in garlic and offers potential genetic targets for enhancing the resilience of this important crop.

## 2. Results

### 2.1. Identification of JAZ Family Members

To identify the *JAZ* gene family in garlic, the JAZ protein sequences from *A. thaliana* were used to construct a hidden Markov model (HMM). A total of 28 *JAZ* genes were identified in the garlic genome and designated as *AsJAZ1* to *AsJAZ28* according to their chromosomal positions (Table 1). The predicted AsJAZ proteins range from 107 (AsJAZ27) to 349 (AsJAZ19) amino acids (aa), with calculated isoelectric points (pI) of 4.94 (AsJAZ20)–10.41 (AsJAZ27) and molecular weights of 12.39 (AsJAZ27)–39.80 (AsJAZ19) kDa. The instability indices vary between 38.32 (AsJAZ26) and 69.77 (AsJAZ18), while the aliphatic indices range from 59.62 (AsJAZ18) to 89.61 (AsJAZ1). The grand average of hydropathicity (GRAVY) values are between −0.984 (AsJAZ20) and −0.155 (AsJAZ1), indicating hydrophilic properties. Subcellular localization predictions suggest that eight AsJAZ proteins are localized in the cytoplasm and 20 in the nucleus.

### 2.2. Phylogenetic Analysis

**Figure 1:**
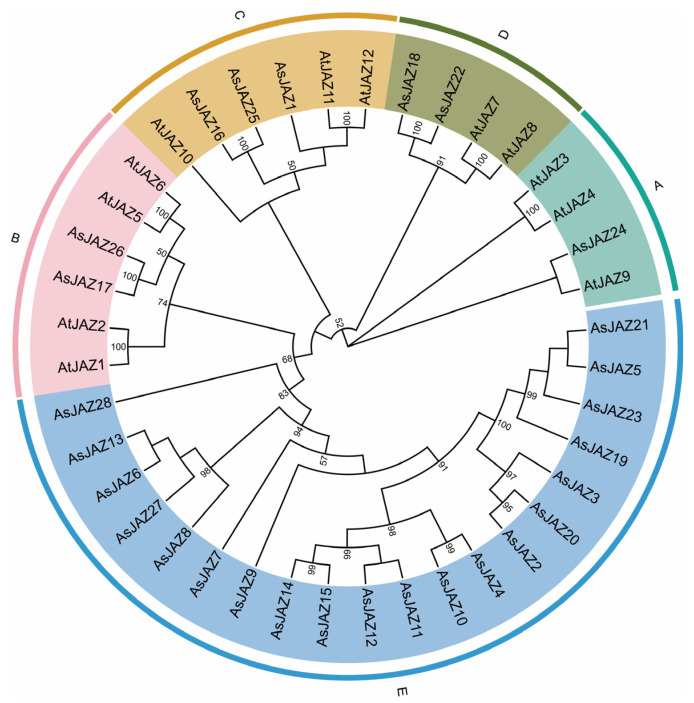
Phylogenetic analysis of the *JAZ* gene family in *A. sativum*. An unrooted neighbor-joining tree was constructed in MEGA 11.0 using the full-length amino acid sequences of the AsJAZ and AtJAZ proteins, the Poisson substitution model, and pairwise deletion of gaps. Bootstrap values from 1000 replicates (shown at nodes) ≥ 50% are indicated. Based on sequence homology, the JAZ proteins cluster into 5 distinct subfamilies (A–E), each marked by a distinct colored arc and shaded background.

A phylogenetic tree was constructed based on the amino acid sequences of 12 *A. thaliana* and 28 garlic JAZ proteins (Figure 1). According to phylogenetic clustering with Arabidopsis thaliana counterparts, the 28 AsJAZ proteins were assigned to 5 distinct subfamilies: Group A–E (containing 1, 2, 3, 2, and 20 members, respectively). All identified AsJAZ proteins clustered into these established subfamilies, with none designated as unclassified. Among the defined subfamilies, all five—A, B, C, D, and E—harbor garlic JAZ members. Notably, 20 (71.43%) of the AsJAZ proteins belong to subfamily E, a clade dominated by garlic JAZs lacking Arabidopsis orthologs, indicating that this specific group has likely undergone significant lineage-specific expansion and may represent the primary functional clade for the JAZ family in garlic.

### 2.3. Conserved Motif and Gene Structure Analysis

**Figure 2:**
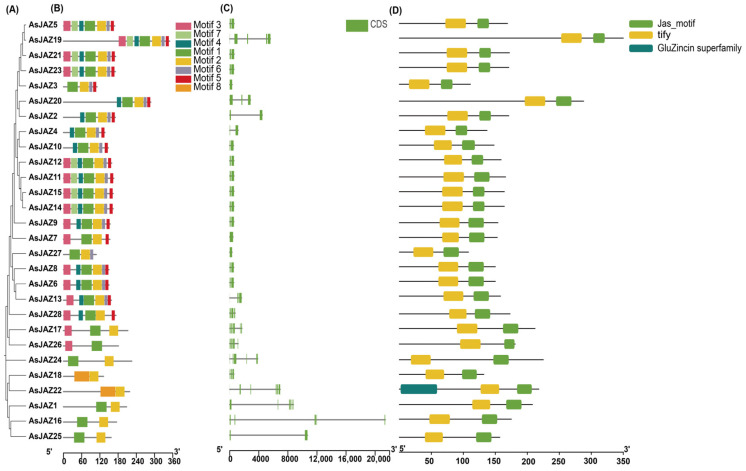
Phylogenetic relationship, conserved protein motifs, gene structure, and domain compositions of *AsJAZ* genes. (**A**): The neighbor-joining (NJ) phylogenetic tree of AsJAZ proteins. (**B**): Distribution of conserved motifs in AsJAZ proteins identified by MEME analysis. The eight identified motifs are represented by different colored boxes (Motif 1–8), and their relative positions are indicated. The scale bar at the bottom represents the length of the protein (aa). (**C**): Exon–intron organization of *AsJAZ* genes. Green boxes represent coding sequences (CDS) and black lines represent introns. The scale bar at the bottom indicates the gene length (bp). (**D**): Conserved domain architecture of AsJAZ proteins. The TIFY domain, Jas_motif, and GluZincin superfamily are indicated by yellow, light green, and dark teal boxes, respectively. The scale bar at the bottom indicates the protein length (aa).

To explore the structural diversity and conservation of AsJAZ proteins, the phylogenetic relationships among the family members were first examined (Figure 2A). Based on this phylogenetic framework, eight conserved motifs were identified and designated as Motif 1–8 (Table S1). The results showed that most AsJAZ members within the same phylogenetic clade (Figure 2A) shared similar motif compositions (Figure 2B). For instance, Motifs 1, 2, 3, 4, and 5 were highly conserved across the majority of the family members, suggesting their essential roles in the fundamental functions of JAZ proteins. However, certain members exhibited distinct motif patterns; for example, AsJAZ19 and AsJAZ20 possessed unique motif arrangements compared to other clades, reflecting potential functional divergence during evolution.

The exon–intron organization of *AsJAZ* genes was analyzed to further understand their evolutionary history (Figure 2C). The number of exons varied among family members, typically ranging from 1 to 6. Most *AsJAZ* genes displayed a relatively simple structure with a limited number of introns. Notably, the total genomic length of these genes showed significant variation; while most members occupied a range within 4000 bp, certain genes such as *AsJAZ16* and *AsJAZ25* contained substantially longer intronic regions, exceeding 10,000 bp. Despite these variations in length, the gene structures remained largely consistent within specific subfamilies.

Domain analysis further confirmed the identity of the AsJAZ proteins (Figure 2D). Almost all identified AsJAZ proteins contained the characteristic TIFY domain (yellow) and the Jas motif (green), which are the defining features of the JAZ (TIFY) family. In most cases, the TIFY domain was located towards the N-terminus relative to the Jas motif, which was consistently positioned near the C-terminus. Interestingly, AsJAZ22 was found to harbor an additional GluZincin superfamily domain at its N-terminus, a feature absent in other members. This unique domain architecture suggests that AsJAZ22 may have specialized regulatory roles distinct from the core JAZ signaling pathway.

### 2.4. Chromosomal Localization

**Figure 3:**
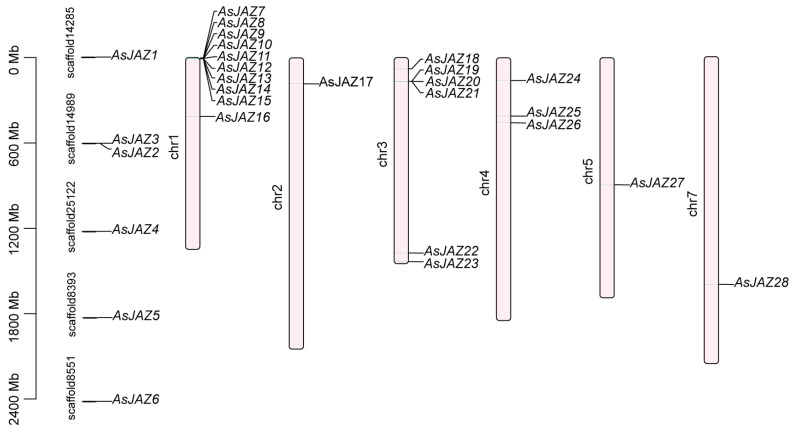
Genomic localization of the *AsJAZ* gene family in *A. sativum*. The physical coordinates of the *AsJAZ* genes are depicted on unanchored scaffolds (positioned on the left) and the six corresponding assembled garlic chromosomes (chr1–chr5, and chr7; positioned on the right). Specific scaffold identifiers are indicated next to their respective sequences, while each individual *AsJAZ* gene is marked according to its approximate genomic mapping. The reference scale bar on the left displays the physical distance in megabases (Mb).

Chromosomal localization analysis revealed that 22 *AsJAZ* genes are distributed across six garlic chromosomes (chr1–chr5, and chr7), while the remaining 6 genes are located on 5 unanchored scaffolds (Figure 3). The number of mapped *AsJAZ* genes per chromosome ranges from 1 to 10. Chromosome 1 harbors the largest cluster, with 10 genes (accounting for approximately 45.45% of the chromosome-mapped members), whereas chromosomes 2, 5, and 7 contain the fewest, with only one gene each (*AsJAZ17*, *AsJAZ27*, and *AsJAZ28*, respectively). Many *AsJAZ* genes are situated in the distal or subtelomeric regions of the chromosomes. The pronounced, dense clustering of *AsJAZ* genes on chromosome 1 (specifically *AsJAZ7* to *AsJAZ16*) and chromosome 3 did not originate from proximal or tandem duplication events according to our collinearity analysis. Instead, this distribution pattern might be the result of ancient WGD events followed by extensive chromosomal rearrangements, which contributed to the current architecture of the *JAZ* family in garlic.

### 2.5. Interspecific Collinearity Analysis

**Figure 4:**
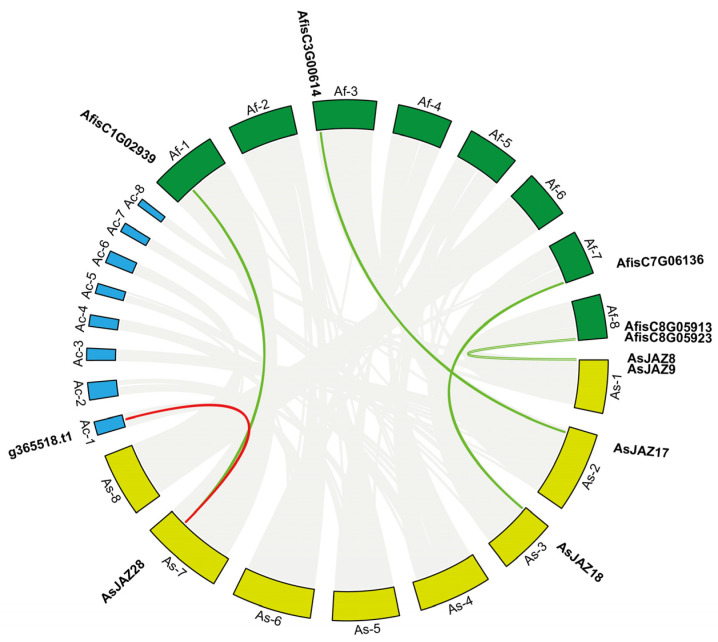
Collinearity analysis of *JAZ* genes among *A. sativum*, *A. fistulosum*, and *A. cepa*. The chromosomes/contigs of *A. fistulosum* (Af), *A. sativum* (As), and *A. cepa* (Ac) are represented by green, yellow, and blue segments, respectively. Gray ribbons in the background indicate the overall syntenic blocks between the genomes. The highlighted colored lines (green and red) represent the orthologous relationships between specific *JAZ* gene pairs across the three species. Gene IDs for orthologous pairs in Af and Ac are labeled alongside their respective chromosomal locations.

To investigate the evolutionary relationship and genomic conservation of *JAZ* genes, a comparative synteny analysis was performed among three Allium species: *A. sativum* (As), *Allium. fistulosum* (Af), and *Allium. cepa* (Ac). The circular map illustrates extensive syntenic blocks across the three genomes, reflecting a high degree of evolutionary conservation within the *Allium* genus (Figure 4).

Several orthologous *JAZ* gene pairs were identified across the species. Notably, *AsJAZ8* and *AsJAZ9*, located on chromosome As-1, exhibited strong syntenic relationships with the *AfisC8G05913* and *AfisC8G05923* loci on Af-8. Similarly, *AsJAZ17* on As-2 was orthologous to *AfisC7G06136* on Af-7, while *AsJAZ18* on As-3 showed a syntenic link to *AfisC3G00614* on Af-3.

Furthermore, *AsJAZ28* on chromosome As-7 demonstrated a conserved syntenic relationship spanning all three species, connecting to *AfisC1G02939* on Af-1 (green line) and the *g365518.t1* locus on Ac-1 (red line). These results indicate that several *JAZ* family members have been highly conserved during the divergence of garlic, Welsh onion, and onion, suggesting that these orthologous pairs may have originated from a common ancestor and potentially maintained similar biological functions.

### 2.6. Analysis of Cis-Acting Elements in Promoter Regions

**Figure 5:**
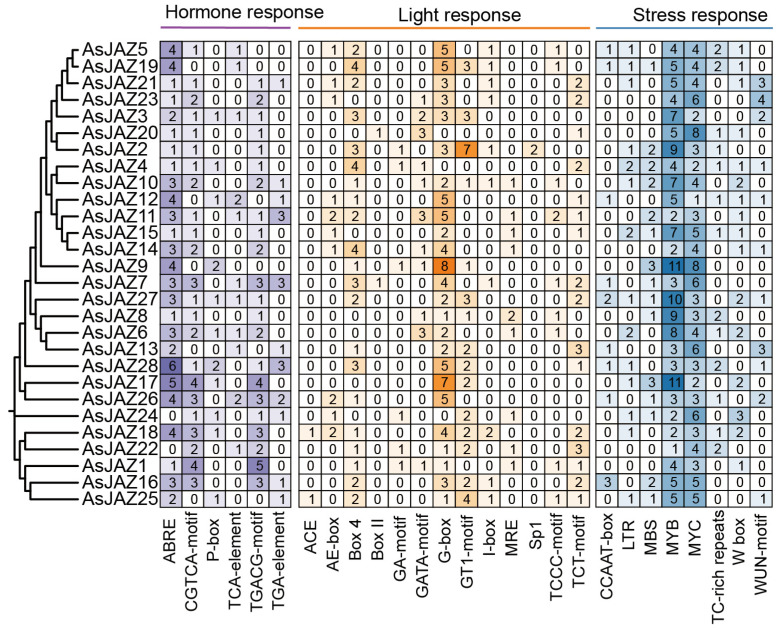
Distribution of cis-acting regulatory elements in the promoters of *AsJAZ* genes. The heatmap illustrates the quantity of predicted regulatory motifs identified within the 2-kb upstream promoter regions of the 28 *AsJAZ* genes. The cis-elements are grouped into three distinct functional categories: hormone response (shaded purple), light response (shaded orange), and stress response (shaded blue). The numbers within each cell, along with the corresponding background color intensity, denote the absolute count of a specific element in each gene’s promoter. A phylogenetic tree is displayed on the left to show the evolutionary clustering of the *AsJAZ* family members.

The 2 kb upstream promoter sequences of the *AsJAZ* genes were investigated to identify predicted cis-acting regulatory elements (Figure 5). These identified motifs were subsequently classified into three primary functional categories: hormone response, light response, and stress response. While each promoter exhibit diverse combinations of these regulatory motifs, their frequencies differ significantly among the family members, indicating divergent transcriptional regulatory profiles.

Abscisic acid-responsive elements (ABRE) and methyl jasmonate-associated motifs (CGTCA-motif and TGACG-motif) are widespread across the family. Promoters of specific genes, such as *AsJAZ28*, *AsJAZ17*, and *AsJAZ9*, display remarkably high concentrations of ABRE or MeJA-related sites, suggesting robust sensitivity to ABA and jasmonic acid signaling pathways. Core light-responsive elements, predominantly G-box and Box 4 motifs, are also extensively distributed. Notably, the promoters of *AsJAZ9*, *AsJAZ17*, and *AsJAZ2* harbor particularly high numbers of G-box sites, implying substantial regulation by light stimuli.

Among the stress-responsive elements—which encompass low-temperature responsiveness (LTR), drought inducibility (MBS), and defense-related components—MYB and MYC transcription factor binding sites are overwhelmingly predominant. Furthermore, several elements explicitly associated with biotic stress and mechanical wounding were identified within this category, including the wound-responsive WUN-motif, the pathogen defense-related W-box, and TC-rich repeats. Specifically, *AsJAZ9*, *AsJAZ27*, and *AsJAZ13* feature exceptional abundance in MYB or MYC binding domains, underscoring their likely fundamental roles in adaptations to varied environmental stresses, while the coexistence of biotic stress-related motifs indicates that these genes also maintain their canonical roles in JA-dependent defense responses against biological threats. The accompanying hierarchical clustering aligns the genes based on shared phylogenetic and regulatory motif profiles; genes like *AsJAZ7* and *AsJAZ27* share a comprehensive, multi-responsive repertoire, whereas others, such as *AsJAZ22*, present relatively simpler element arrays, potentially indicating a more specialized regulatory scope.

### 2.7. Expression Pattern Analysis

**Figure 6:**
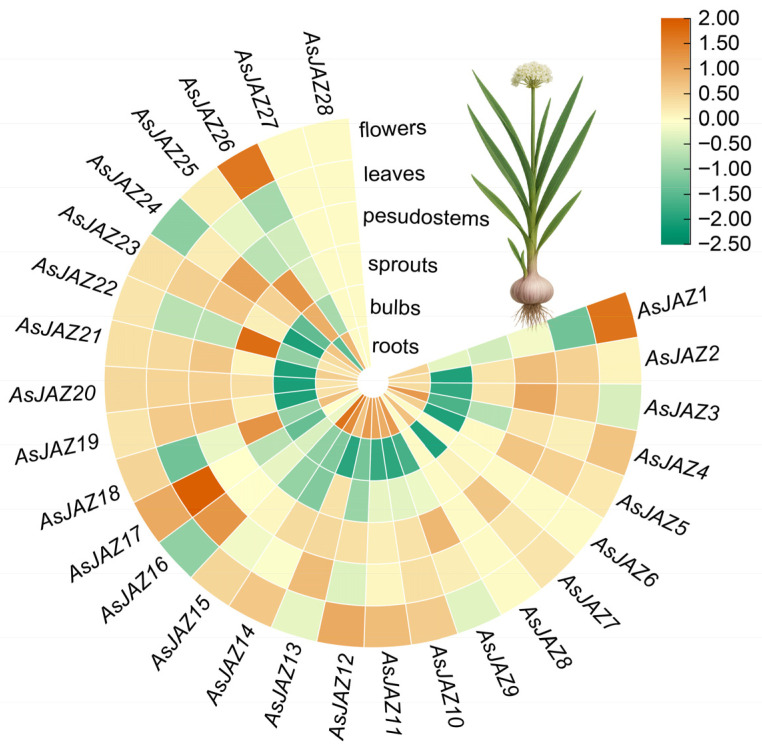
Tissue-specific expression profiles of the *AsJAZ* gene family in *A. sativum*. The circular heatmap illustrates the relative transcript abundance of the 28 *AsJAZ* genes across six distinct garlic tissues: roots, bulbs, sprouts, pseudostems, leaves, and flowers. The color gradient represents normalized expression levels, with green indicating lower transcript abundance and orange indicating higher transcript abundance. The corresponding scale bar is provided on the top right.

Transcriptome analysis across six tissues revealed differential expression patterns among the 28 *AsJAZ* genes (Figure 6). Overall, the highest transcript accumulation is predominantly observed in flowers, leaves, and roots, suggesting that these organs serve as primary functional domains for JAZ-mediated signaling and development in garlic.

At the individual gene level, distinct tissue-preferential signatures can be observed. In reproductive tissues, *AsJAZ1*, *AsJAZ17*, and *AsJAZ26* exhibit prominent up-regulation within floral organs. Meanwhile, in aerial vegetative tissues, transcript accumulation peaks in the leaves for *AsJAZ13*, *AsJAZ16*, and *AsJAZ17*, whereas *AsJAZ3*, *AsJAZ9*, and *AsJAZ24* display their highest expression levels within the pseudostems.

Evaluation of spatial distribution further highlights specific associations within below-ground and storage organs. A distinct group of genes—including *AsJAZ4*, *AsJAZ14*, and *AsJAZ15*—shows preferential high expression predominantly restricted to the roots. Within the bulbs, *AsJAZ25* exhibits notably high transcript abundance. Additionally, in the sprouts, *AsJAZ18*, *AsJAZ22*, and *AsJAZ25* demonstrate peak expression levels. Conversely, a specific subset of genes—comprising *AsJAZ6*, *AsJAZ8*, *AsJAZ27*, and *AsJAZ28*—exhibits zero or negligible expression across all the analyzed tissues under standard growth conditions. This basal suppression implies that these members may be subjected to strict transcriptional control, potentially serving as specialized regulatory components that are exclusively inducible upon environmental stresses or specific hormone elicitation.

### 2.8. qRT–PCR Analysis

**Figure 7:**
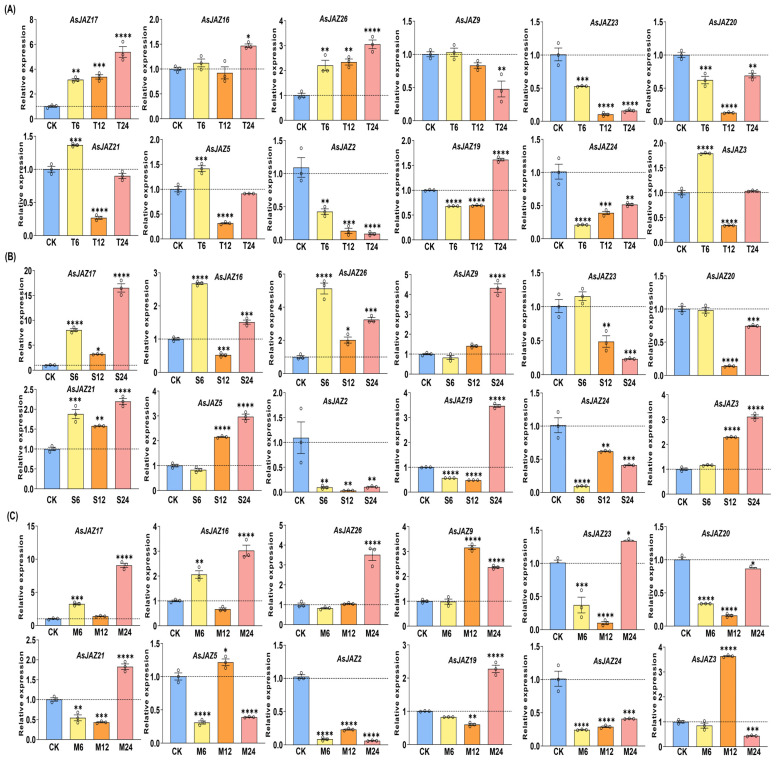
qRT–PCR analysis of selected *AsJAZ* genes in garlic under various stress treatments. Seedlings were subjected to (**A**) heat stress (39 °C), (**B**) salt stress (200 mM NaCl), or (**C**) methyl jasmonate treatment (100 µM MeJA) for 6, 12, and 24 h. “CK” denotes the untreated control plants. Data are presented as the mean ± standard deviation (SD) of three biological replicates (n = 3), with each biological replicate comprising three technical replicates. Asterisks indicate significant differences relative to the CK group at each time point, determined by one-way analysis of variance (ANOVA) followed by Duncan’s multiple range test (* p < 0.05, ** p < 0.01, *** p < 0.001, **** p < 0.0001).

To validate the stress-responsive expression patterns of *AsJAZ* genes observed in transcriptomic analysis and clustering, we selected twelve representative members (*AsJAZ17*, *-16*, *-26*, *-9*, *-23*, *-20*, *-21*, *-5*, *-2*, *-19*, *-24*, and *-3*) for quantitative real-time PCR (qRT–PCR) (Figure 7). These genes were chosen based on their expression profiles and their potential differential responsiveness under various treatments. These treatments were selected to represent the major abiotic environmental challenges in garlic production (heat and salt) and to evaluate the core response of *AsJAZ* genes to the jasmonate signaling pathway (MeJA). Leaves are typically the primary sites of physiological and phenotypic changes during environmental stress, making them an ideal tissue for identifying stress-related gene activity. Garlic plants were subjected to heat (represented by T), salt (S), and methyl jasmonate (M) treatments, and leaf samples were collected at 0, 6, 12, and 24 h for analysis.

Under heat stress (Figure 7A), *AsJAZ17* and *AsJAZ26* were significantly induced, with transcript levels increasing and peaking at 24 h. *AsJAZ21* and *AsJAZ5* showed an early response, with expression peaking at 6 h before declining. Conversely, *AsJAZ2*, *AsJAZ23*, and *AsJAZ24* displayed progressive downregulation over the treatment period, indicating that these genes may be repressed by high temperatures or function predominantly under non-stress conditions. Under salt treatment (Figure 7B), *AsJAZ17*, *AsJAZ9*, and *AsJAZ19* showed strong induction at 24 h, with *AsJAZ17* reaching a particularly notable expression level. *AsJAZ16* and *AsJAZ26* exhibited early induction at 6 h. In contrast, *AsJAZ2*, *AsJAZ24*, and *AsJAZ20* exhibited persistent downregulation throughout the salt exposure, implying either a functional suppression by salinity or involvement in different regulatory pathways. Following MeJA application (Figure 7C), *AsJAZ17*, *AsJAZ26*, *AsJAZ9*, and *AsJAZ19* were upregulated at later time points (12 h or 24 h). *AsJAZ3* displayed a distinct and specific induction at 12 h. Notably, *AsJAZ2* and *AsJAZ24* again showed sustained downregulation under MeJA treatment, exhibiting persistent repression across all three stresses, consistent with potential roles as negative regulators or stress-suppressed factors.

Together, these qRT–PCR results confirm that most selected *AsJAZ* genes are robustly responsive to heat, salt, and MeJA, with *AsJAZ17* and *AsJAZ26* emerging as versatile multi-stress responsive markers. Meanwhile, the downregulated subset may participate in energy reallocation or negative feedback during stress responses.

### 2.9. Protein–Protein Interaction Network and Gene Ontology Enrichment

**Figure 8:**
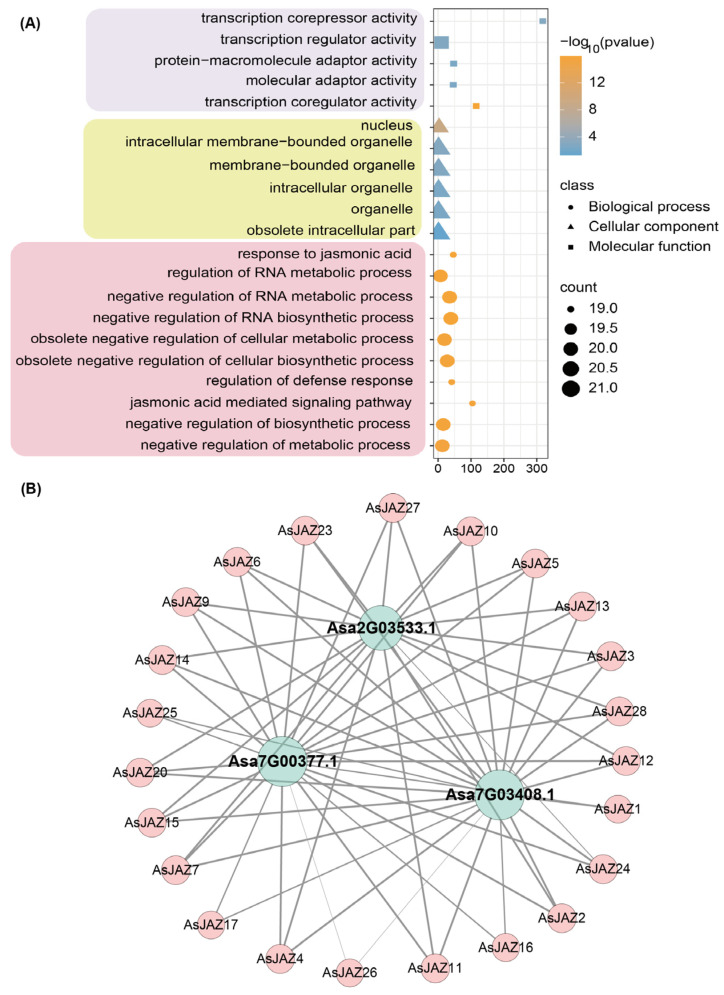
GO enrichment and interaction network of AsJAZ proteins. (**A**) GO enrichment analysis. Terms are categorized into Molecular Function (squares), Cellular Component (triangles), and Biological Process (circles). Node size represents gene count; color reflects significance (–log_10_(*p*-value)). (**B**) Predicted PPI network. Peripheral pink nodes represent AsJAZ proteins. Central light blue nodes are key interacting hub proteins. Grey lines denote predicted interactions.

Gene Ontology analysis (Figure 8A) revealed that the *AsJAZ* genes are significantly enriched in biological processes (BP) associated with the “response to jasmonic acid,” the “jasmonic acid mediated signaling pathway,” and the “negative regulation of RNA biosynthetic process.” Within the cellular component (CC) category, these genes are predominantly mapped to the “nucleus” and “intracellular membrane-bounded organelle,” consistent with their anticipated roles as nuclear-localized components. For molecular function (MF), the most highly enriched terms include “transcription corepressor activity” and “transcription regulator activity.” Together, these functional annotations align closely with the established roles of JAZ proteins as transcriptional repressors in plant hormone signaling.

To further map the functional relationships of these proteins, a predictive PPI network was constructed (Figure 8B). The network displays a distinct topology wherein the majority of the peripheral AsJAZ proteins (colored pink) interact directly with three central hub proteins (colored blue). Based on annotation, the hub gene *Asa2G03533.1* encodes a coronatine-insensitive protein 1 (COI1) homolog, which functions as a primary jasmonate receptor. The other two central nodes, *Asa7G00377.1* and *Asa7G03408.1*, are identified as MYC2 transcription factors. The extensive, high-confidence interactions between the diverse AsJAZ family members and these specific hub proteins indicate that garlic possesses a conserved core JA signaling module (COI1-JAZ-MYC2), suggesting that AsJAZ proteins function by physically complexing with COI1 and MYC2 to mediate downstream transcriptional responses.

### 2.10. Subcellular Localization and Protein–Protein Interaction Analysis of *AsJAZs*

**Figure 9:**
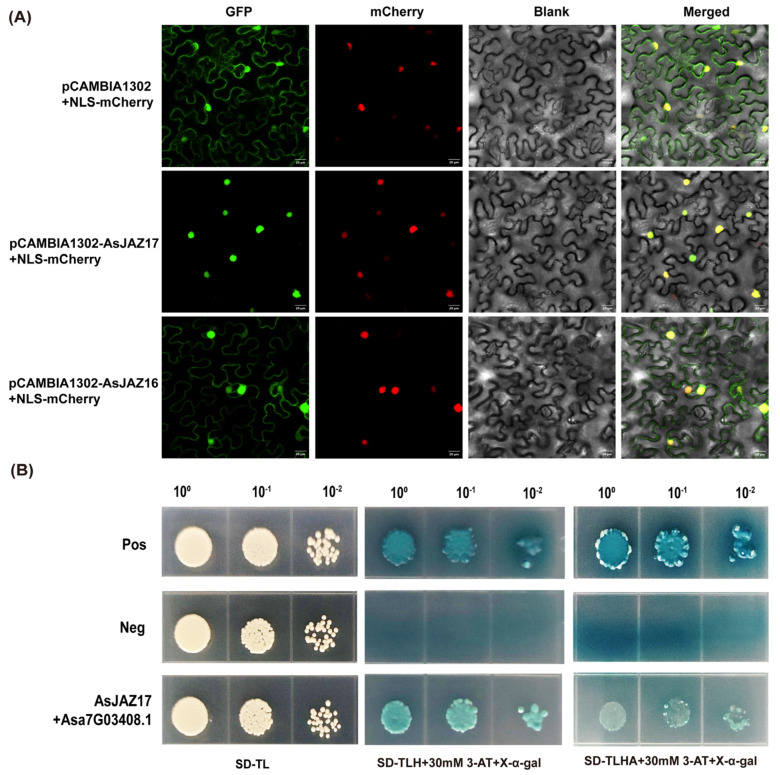
Subcellular localization and protein–protein interaction analyses of AsJAZ proteins. (**A**) Subcellular localization of AsJAZ16 and AsJAZ17. Transient co-expression of the GFP fusion proteins (AsJAZ16-GFP and AsJAZ17-GFP) and the empty vector (pCAMBIA1302-GFP) with the nuclear marker NLS-mCherry in *N. benthamiana* leaf epidermal cells. The panels from left to right represent GFP fluorescence (green), mCherry fluorescence (red), bright-field images (Blank), and merged images. The free GFP signal from the empty pCAMBIA1302 vector is distributed in both the cytoplasm and the nucleus. The AsJAZ17-GFP signal strictly colocalizes with the nuclear marker NLS-mCherry, whereas the AsJAZ16-GFP signal is detected in both the cytoplasm and the nucleus. Scale bars = 20 μm. (**B**) Y2H assay confirming the interaction between AsJAZ17 and the MYC2 transcription factor Asa7G03408.1. Yeast cells co-expressing the indicated constructs were spotted onto non-selective medium (left panels) and selective media supplemented with X-α-Gal (middle and right panels) in a series of 10-fold dilutions (10^0^, 10^−1^, 10^−2^). Pos represents the positive control; Neg represents the negative control.

To validate the bioinformatic predictions and investigate the cellular compartments where AsJAZ proteins exert their biological functions, AsJAZ16 and AsJAZ17 were selected as representative candidates due to their distinct predicted localization patterns and their robust transcriptional induction under salt stress (Figure 7B). We performed transient expression assays in *Nicotiana benthamiana* leaf epidermal cells (Figure 9A). GFP-fused constructs of AsJAZ16 and AsJAZ17 were co-expressed with the nuclear marker NLS-mCherry to determine their subcellular distribution. Confocal imaging of the empty-vector control (pCAMBIA1302) showed that the free GFP signal was ubiquitously distributed throughout the cell, including the cytoplasm and the nucleus. Under the same experimental conditions, the AsJAZ17-GFP fluorescence was concentrated exclusively within the nucleus and overlapped precisely with the NLS-mCherry signal, indicating a strict nuclear localization. In contrast, AsJAZ16-GFP exhibited a broader distribution pattern; the green fluorescence signal was observed both within the nucleus, where it colocalized with NLS-mCherry, and in the cytoplasm along the cell periphery and transvacuolar strands. These observations demonstrate that AsJAZ16 and AsJAZ17 possess distinct subcellular localization patterns, with AsJAZ17 being a nuclear protein and AsJAZ16 being localized to both the nucleus and the cytoplasm. Such differential partitioning suggests that these two JAZ proteins may fulfill different regulatory roles within their respective cellular environments.

To further validate the functional relationships predicted by our PPI network, we performed a yeast two-hybrid (Y2H) assay to test the interaction between the strictly nuclear-localized AsJAZ17 and the predicted central hub protein Asa7G03408.1, a MYC2 transcription factor. As shown in Figure 9B, yeast cells co-transformed with *AsJAZ17* and *Asa7G03408.1* grew normally and developed blue colonies on the selective dropout media, exhibiting a phenotype similar to the positive control. In contrast, the negative control strains failed to survive under the same selective conditions. These results provide direct in vivo evidence that AsJAZ17 physically interacts with the MYC2 transcription factor Asa7G03408.1, firmly corroborating the bioinformatic predictions and supporting the existence of a conserved COI1-JAZ-MYC2 signaling module in garlic.

### 2.11. Overexpression of AsJAZ17 Enhances Salt Tolerance in Transgenic Arabidopsis

**Figure 10:**
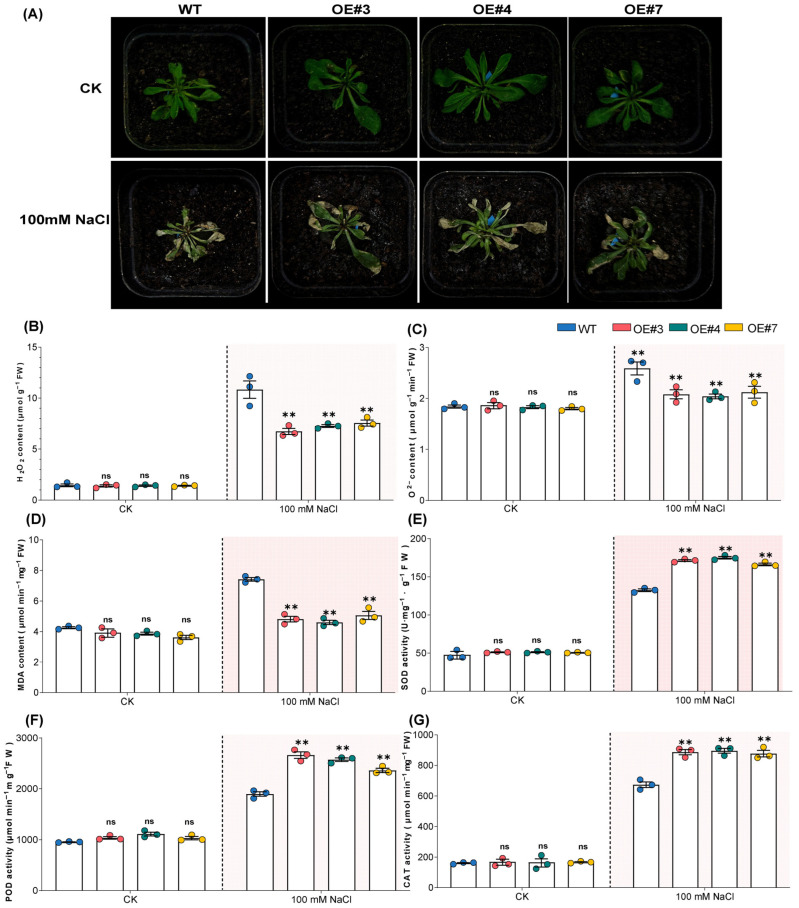
Phenotypic and physiological responses of wild-type (WT) and transgenic lines under salt stress. (**A**) Growth phenotypes of WT and transgenic lines (OE#3, OE#4, and OE#7) under control (CK) and salt stress (100 mM NaCl) conditions. Images were captured after 72 h of treatment. (**B**–**D**) Concentration of ROS and lipid peroxidation markers: (**B**) H_2_O_2_ content, (**C**) O_2_^−^ content, and (**D**) MDA content. (**E**–**G**) Activities of antioxidant enzymes: (**E**) SOD, (**F**) POD and (**G**) CAT. All physiological parameters were measured in the leaves of 4-week-old plants. Data are presented as the mean ± SD (n = 3). Asterisks indicate significant differences between WT and OE lines under the same treatment according to Student’s *t*-test (** *p* < 0.01; ns, not significant).

To evaluate the biological role of the *AsJAZ* family in stress adaptation, we prioritized *AsJAZ17* as the primary candidate for functional validation. This choice was driven by its exceptionally robust transcriptional induction under salinity—which was significantly more pronounced than that of other members such as *AsJAZ16* (Figure 7B)—as well as the high density of salt-responsive elements in its promoter (Figure 5) and its central hub status in the predicted PPI network (Figure 8B). While *AsJAZ16* also exhibited salt-responsiveness, its potential functional role will be further explored in our future research to provide a more comprehensive understanding of the family’s diversity. In the present study, we observed the growth phenotypes of wild-type (WT) and three independent *AsJAZ17*-overexpressing lines (OE#3, OE#4, and OE#7). Under control conditions (CK), no significant morphological differences were observed between the WT and OE lines. However, upon exposure to 100 mM NaCl for 72 h, the WT plants exhibited severe growth inhibition, characterized by evident leaf wilting and chlorosis. In contrast, the *AsJAZ17*-OE lines displayed a more robust growth phenotype with higher biomass and reduced leaf damage compared to the WT (Figure 10A). The levels of ROS and MDA were measured to assess the degree of oxidative stress and membrane damage. Under control conditions, there were no significant differences in H_2_O_2_, superoxide anion (O_2_^−^) and malondialdehyde (MDA) contents between the WT and OE lines. Salt stress (100 mM NaCl) led to a marked increase in ROS accumulation in all lines; however, the levels of ROS in the OE lines were significantly lower than those in the WT (Figure 10B,C). Similarly, the MDA content, a key indicator of lipid peroxidation, was significantly lower in the transgenic lines than in the WT under salt treatment (Figure 10D). These results suggest that *AsJAZ17* overexpression reduces salt-induced oxidative damage in Arabidopsis. To further investigate the mechanism by which *AsJAZ17* regulates ROS homeostasis, the activities of major antioxidant enzymes, including Superoxide dismutase (SOD), Peroxidase (POD), and catalase (CAT), were analyzed. Under normal growth conditions, SOD, POD, and CAT activities remained at basal levels with no significant differences between genotypes. Following salt treatment, the activities of these three enzymes were significantly up-regulated in all plants. Notably, the OE lines exhibited significantly higher SOD, POD, and CAT activities compared to the WT plants (Figure 10E–G). These findings indicate that the enhanced salt tolerance in *AsJAZ17*-OE lines is associated with an improved antioxidant defense system.

## 3. Discussion

JAZ proteins are core repressors in the JA signaling pathway, playing crucial roles in regulating plant vegetative growth, reproductive development, and responses to various biotic and abiotic stresses. In this study, a total of 28 *AsJAZ* genes were identified in the garlic genome. Compared to the *JAZ* family sizes in other well-studied model plants or crops, such as *Arabidopsis thaliana* (12), maize (*Zea mays*) (16), and rice *(Oryza sativa)* (23), garlic possesses a significantly larger repertoire of *JAZ* members [14,15,16]. Our chromosomal localization analysis revealed an uneven distribution of *AsJAZ* genes, with pronounced, dense clustering observed on specific chromosomes, particularly chromosome 1 (harboring 10 genes, roughly 45%) and chromosome 3. This highly clustered genomic arrangement on chromosome 1 might not be attributed to recent tandem duplications, as our collinearity analysis detected no such events. Instead, it likely reflects ancient whole-genome duplication events followed by complex chromosomal rearrangements and lineage-specific retentions, contributing to the expansion of the *AsJAZ* family in garlic. Phylogenetic analysis further elucidated the complex evolutionary trajectory of AsJAZs. The AsJAZ proteins clustered into five distinct subfamilies (A–E). Strikingly, 20 out of 28 AsJAZ members (71.43%) were entirely assigned to Subfamily E, a massive clade exclusively composed of garlic JAZs without any *Arabidopsis* orthologs. This phenomenon indicates a significant lineage-specific expansion within the garlic JAZ family. Lineage-specific expanded genes are often retained during evolution to facilitate adaptation to specific ecological niches or to govern unique developmental processes [17,18]. Consistent with this, our transcriptomic and qRT-PCR data demonstrate that multiple Subfamily E genes are induced by abiotic stress and MeJA treatment. Tissue-specific expression profiles indicate that certain members, such as *AsJAZ14* and *AsJAZ15*, are preferentially expressed in roots, suggesting a role in localized stress perception. The expansion of Subfamily E introduces functional redundancy into the gene family. This redundancy may function as a regulatory buffer to modulate the intensity of jasmonate signaling, assisting the plant in maintaining the growth-defense trade-off during environmental stress. Despite the rapid expansion and diversification of certain clades, core elements of the *JAZ* family remain highly conserved. Interspecies collinearity analysis among three *Allium* species revealed extensive syntenic blocks and several highly conserved orthologous *JAZ* gene pairs, such as *AsJAZ28*. This high syntenic conservation typically implies that these orthologs have been subjected to strong purifying selection to maintain fundamental biological functions in the JA signaling pathway across the *Allium* genus.

The biological function of JAZ proteins is intrinsically linked to their conserved domains and precise subcellular distribution. In this study, domain analysis confirmed that nearly all 28 AsJAZ proteins harbor the characteristic TIFY and Jas motifs. The TIFY domain is essential for repressor complex formation, while the Jas motif mediates interaction with COI1 receptors and MYC transcription factors [19,20]. Notably, AsJAZ22 was found to possess a unique gluzincin superfamily domain at its N-terminus. This distinct domain architecture suggests that AsJAZ22 may have evolved specialized regulatory roles or participated in crosstalk between JA signaling and proteolytic pathways [21,22]. Furthermore, the unusually substantial intronic regions observed in *AsJAZ16* and *AsJAZ25* (>10,000 bp) point toward complex transcriptional regulation, such as alternative splicing or the presence of internal regulatory elements. Subcellular localization is a critical determinant of protein function. Our experimental results for AsJAZ17 are consistent with the established model of JAZs as nuclear-localized transcriptional repressors, aligning with its predicted role in the COI1-JAZ-MYC2 signaling module identified in the PPI network. However, the distinct localization patterns of AsJAZ16 and AsJAZ17 suggest a refined layer of functional specialization within the garlic JAZ family. While JAZ proteins are traditionally characterized as nuclear-localized components, our findings reveal that AsJAZ16 exhibits a nucleocytoplasmic distribution. This pattern is consistent with observations in other plant species, such as *Lycoris aurea*, where certain JAZ members have also been identified to localize within the cytoplasm or exhibit nucleocytoplasmic co-localization [23]. Crucially, our subcellular localization predictions indicate that 8 out of the 28 identified AsJAZ members are not exclusively localized to the nucleus. This distribution suggests that the spatial partitioning and potential nucleocytoplasmic shuttling observed for AsJAZ16 represent a broader regulatory mechanism within the garlic JAZ family, rather than an isolated case. The presence of AsJAZ16, and potentially other members, in both the cytoplasm and the nucleus implies a more complex and dynamic regulatory mechanism, possibly involving cytoplasmic sequestration. Sequestering a portion of JAZ proteins in the cytoplasm may provide a cellular reservoir. Such a strategy would allow the plant to fine-tune the intensity and duration of JA responses by modulating the concentration of the repressor available to interact with nuclear transcription factors in response to specific developmental or environmental cues. This spatial divergence underscores the functional compartmentalization within the garlic JAZ family; while AsJAZ17 functions as a direct nuclear repressor, AsJAZ16 may participate in non-genomic JA signaling processes in the cytoplasm or act as a scaffold protein to integrate JA signaling with other cytoplasmic pathways before translocating into the nucleus. This multi-layered regulatory architecture enables the plant to achieve a more nuanced and rapid response to JA-mediated signals. Collectively, these results indicate that the garlic JAZ family has evolved distinct subcellular partitioning strategies, reflecting a sophisticated evolutionary adaptation to ensure a highly coordinated and flexible defense and developmental program through diverse cellular environments. The expression profile of a gene is fundamentally linked to the cis-acting regulatory elements within its promoter region. In this study, transcriptome analysis revealed that a substantial proportion of *AsJAZ* genes exhibit preferential transcript accumulation in the roots. Given that roots serve as the primary interface for sensing soil-borne abiotic stresses, this suggests their involvement in early stress perception and JA signal relay. The robust induction of *AsJAZ17*, *AsJAZ26*, and *AsJAZ9* under heat, salt, and MeJA treatments correlates with an abundance of ABRE, MeJA-responsive motifs, and MYB/MYC binding sites in their promoters [24]. Notably, the high frequency of G-box motifs in *AsJAZ9* and *AsJAZ17* implies potential roles in the crosstalk between light signaling and JA-mediated pathways [25]. The qRT–PCR analysis also demonstrated divergent response patterns, characterized by both significant up-regulation and persistent downregulation. Members such as *AsJAZ2* and *AsJAZ24* were consistently repressed following stress exposure. Since JAZ proteins are repressors, their downregulation likely facilitates the release of inhibition on downstream defense genes. Conversely, the strong induction of *AsJAZ17* might serve as a negative feedback mechanism to prevent over-activation of JA signaling. This synergistic action of induction and repression reflects a sophisticated strategy to maintain signal homeostasis and optimize resource allocation under adverse conditions [26]. The promoter region of *AsJAZ17* contains both abscisic acid-responsive elements (ABRE) and MeJA-associated motifs (CGTCA/TGACG-motifs), suggesting its involvement in both signaling pathways. At the molecular level, *AsJAZ17* interacts with MYC2, which serves as a convergence point for hormone crosstalk. This interaction coordinates JA-mediated defense responses with ABA-mediated osmotic adjustment. During salt stress, *AsJAZ17* may prevent JA signaling from antagonizing ABA protective pathways, thereby supporting antioxidant enzyme activity and cellular homeostasis. This coordination between hormones is a strategy for garlic to maintain physiological balance under environmental stress.

The integration of PPI modeling and functional validation identifies AsJAZ17 as a central regulator of the salt stress response in garlic. The predictive PPI network, complemented by our Y2H assay, demonstrates that AsJAZ17 serves as a key node by interacting directly with the core JA signaling machinery, specifically the MYC2 transcription factor (Asa7G03408.1). This confirmed physical interaction establishes that AsJAZ17 operates through the canonical COI1-JAZ-MYC2 signaling module to translate JA signals into downstream transcriptional changes. Beyond this specific interaction, the functional dynamics of the garlic JA signaling network are also influenced by internal interactions among the 28 AsJAZ members. As indicated by the predictive PPI network (Figure S1), the conserved TIFY domain present in these proteins facilitates the formation of various JAZ-JAZ complexes. This structural capability suggests that different JAZ members compete for interaction with central transcription factors, such as MYC2. This competitive binding, combined with the redundancy provided by the expanded Subfamily E, creates a regulatory buffer. This internal network allows the plant to calibrate its physiological responses and maintain signaling homeostasis as individual JAZ proteins undergo stress-induced degradation. Functional evidence from transgenic Arabidopsis lines further supports this role, as *AsJAZ17*-overexpressing (OE) lines exhibited significantly improved growth phenotypes, characterized by higher biomass and reduced chlorosis compared to wild-type (WT) plants under 100 mM NaCl treatment. The observation that overexpressing a transcriptional repressor enhances stress tolerance can be elucidated through the “Growth-Defense Trade-off” mechanism. While the JA signaling pathway is essential for defense, its persistent activation typically leads to severe growth inhibition as the plant reallocates metabolic resources away from primary growth. Specifically, unchecked activity of MYC2—the master activator of JA defense—can negatively regulate growth by inhibiting primary root development and suppressing genes involved in primary metabolism [27,28]. By physically complexing with MYC2, *AsJAZ17* restricts the ability of this transcription factor to promote physiological arrest, thereby preventing the runaway activation of the JA response. This calibration allows the plant to preserve the necessary physiological vigor and metabolic energy required for survival during prolonged salinity. Furthermore, the enhancement of salt tolerance is tightly linked to precise hormonal crosstalk and ROS homeostasis. MYC2 often functions as a central node for antagonistic cross-talk between JA signaling and other stress hormones, such as abscisic acid (ABA). High levels of MYC2 can misdirect the cellular response toward JA-mediated pathways at the expense of ABA-dependent pathways, which are essential for osmotic adjustment and drought/salt resilience [29,30]. By sequestering MYC2 through direct protein–protein interaction, the overexpression of *AsJAZ17* optimizes this hormonal balance, preventing it from antagonizing ABA-mediated protective mechanisms. This targeted regulation directly contributes to a highly efficient ROS-scavenging machinery; consequently, the *AsJAZ17*-OE lines showed significantly lower levels of H_2_O_2_, O_2_^−^, and MDA, supported by markedly higher activities of SOD, POD, and CAT under stress. At the transcriptional level, this enhancement of antioxidant capacity is intrinsically linked to the JA signaling module. In established JA signaling models, the central basic helix–loop–helix (bHLH) transcription factor MYC2 recognizes and binds to conserved sequence elements, such as G-box motifs. Through this regulatory mechanism, MYC2 coordinates the expression of downstream stress-responsive networks, which include genes encoding ROS-scavenging enzymes like SOD, POD, and CAT. By modulating the availability and transcriptional activity of MYC2, *AsJAZ17* effectively controls the transcription of these antioxidant genes to maintain ROS homeostasis. Beyond simple detoxification, this improved stress resilience involves a sophisticated regulation of the intracellular redox environment. Current models emphasize the role of ROS and reactive nitrogen species (RNS) signaling crosstalk in plant stress adaptation [31]. In this context, the JA signaling module integrates with redox-mediated processes. By regulating the antioxidant defense system, *AsJAZ17* assists in maintaining precise ROS/RNS homeostasis. This controlled regulation prevents oxidative toxicity while permitting these reactive species to function as essential secondary messengers within the broader stress response network. Finally, the increased abundance of AsJAZ17 proteins in transgenic lines provides a crucial “buffer pool” against stress-induced degradation. Because JAZ proteins are degraded via the 26S proteasome upon JA perception, a standard plant may quickly deplete its repressor stock during an environmental JA spike. In the OE lines, the larger reservoir of *AsJAZ17* ensures that a sufficient population of repressors remains functional to maintain cellular homeostasis and prevent oxidative damage from over-active defense signaling. Notably, the phenomenon of *JAZ* gene overexpression enhancing abiotic stress tolerance is not restricted to *Arabidopsis* or garlic; similar findings have been documented in other major crops, including cotton and grape [32,33]. These collective results emphasize that the stabilization of repressors like *AsJAZ17* is a strategic adaptation for fine-tuning environmental resilience, positioning it as a vital candidate for molecular breeding programs aimed at improving the yield stability of garlic cultivars.

Although this study verified the physical interaction between AsJAZ17 and MYC2 in garlic, the precise downstream target genes regulated by this module require further investigation. Based on studies in model plants, MYC2 directly activates a series of jasmonate (JA)-responsive genes, including *VSP2*, as well as key JA biosynthesis genes such as *LOX2*, *AOS*, and *OPR3* [11,34]. In our study, *AsJAZ17* overexpression increased SOD, POD, and CAT activities, indicating that under salt stress, the AsJAZ17–MYC2 module may directly or indirectly regulate these redox homeostasis-related genes. Furthermore, MYC2 can directly activate the transcription of JAZ family members (e.g., *JAZ5*, *JAZ6*, and *JAZ10*) to form a negative feedback loop. This loop is necessary to prevent excessive energy consumption during defense responses and to maintain the growth-defense trade-off.

Consistent with this regulatory role, the robust induction of *AsJAZ17* by heat and MeJA indicates its involvement in a broader stress-responsive network beyond salt tolerance. Given its ability to reinforce the antioxidant defense system, *AsJAZ17* may contribute to resilience against various abiotic and biotic challenges where oxidative stress is a key factor. Specifically, the interaction with the master regulator MYC2 suggests that *AsJAZ17* could act as a ‘molecular brake’ to optimize the growth-defense trade-off during pathogen or herbivore encounters, preventing excessive metabolic resource reallocation. Future research utilizing specific pathogen infection assays on these transgenic lines will be essential to fully elucidate the multi-stress resilience conferred by this module.

However, the resolution of our current findings is limited by the use of bulk tissue transcriptomics. While this approach identifies tissue-preferential expression, it does not account for cell-type-specific heterogeneity. Resolving plant stress responses at cellular resolution is necessary to understand signaling dynamics, as expression and regulation can vary among adjacent cell layers [35]. Applying single-cell and spatial transcriptomics in future studies will provide a required framework to dissect the cellular heterogeneity and spatial regulation of JA-mediated stress responses in garlic.

Although the heterologous *Arabidopsis* system provided initial functional insights into *AsJAZ17*, in planta validation in garlic remains a necessary step for crop improvement. Establishing a stable genetic transformation system for garlic is currently difficult due to its large genome and high proportion of repetitive sequences. Future research will focus on utilizing methods such as virus-induced gene silencing (VIGS) or optimized CRISPR/Cas9 gene-editing protocols to directly evaluate the function of *AsJAZ* genes in garlic cultivars. Modifying or knocking out *AsJAZ17* directly in the garlic background will provide direct evidence of its role in stress regulation and facilitate the molecular breeding of stress-tolerant cultivars.

## 4. Materials and Methods

### 4.1. Genome-Wide Identification of JAZ Genes

Genomic and protein sequences of garlic were retrieved from the AlliumDB database (https://allium.qau.edu.cn/, accessed on 3 March 2025) [36], and Arabidopsis thaliana sequences were obtained from the NCBI database (https://www.ncbi.nlm.nih.gov/, accessed on 3 March 2025). A. thaliana JAZ protein sequences (AtJAZ) were sourced from The Arabidopsis Information Resource (TAIR, version 10, http://www.arabidopsis.org, accessed on 3 March 2025) [37]. A local protein database was established, and BLASTP (v2.11.0) searches (E-value < 1 × 10^−5)^ were conducted using NCBI BLAST+ (v2.11.0) to identify candidate JAZ family members by sequence alignment. The HMM (Hidden Markov Model) profiles of the TIFY domain (PF06200) and the JAS domain (PF09425) were downloaded from the Pfam database (http://pfam-legacy.xfam.org/, accessed on 3 March 2025) and applied with HMMER v3.3.2 (http://hmmer.org/, accessed on 18 March 2025) to further screen potential JAZ proteins [38]. Candidate sequences were validated using SMART 2.8 (http://smart.embl-heidelberg.de/, accessed on 3 March 2025) [39] and the NCBI Conserved Domain Database (CDD, https://www.ncbi.nlm.nih.gov/cdd, accessed on 3 March 2025) [40] to confirm domain integrity. Physicochemical properties—sequence length, molecular weight, theoretical isoelectric point, instability index, aliphatic index and grand average of hydropathicity (GRAVY)—were computed with ProtParam (ExPASy, https://web.expasy.org/protparam/, accessed on 3 March 2025) [41]. Transmembrane regions were predicted using TMHMM 2.0 (DTU Health Tech, https://services.healthtech.dtu.dk/services/TMHMM-2.0/), subcellular localization with Cell-PLoc 2.0 (SJTU, http://www.csbio.sjtu.edu.cn/bioinf/Cell-PLoc-2/, accessed on 3 March 2025) and secondary structure with SOPMA (I-TASSER, https://npsa-prabi.ibcp.fr/cgi-bin/npsa_automat.pl?page=npsa_sopma.html, accessed on 3 March 2025).

### 4.2. Phylogenetic and Gene Structure Analyses

Full-length JAZ protein sequences from garlic and *A. thaliana* were retrieved from UniProt (https://www.uniprot.org/, accessed on 3 March 2025). Multiple sequence alignment was performed with ClustalX 1.81 (http://www.clustal.org/clustal2/, accessed on 5 March 2025) and manually adjusted in Jalview (v2.11.2.5). A neighbor-joining phylogenetic tree was constructed in MEGA 11.0 (https://www.megasoftware.net/) using the Poisson model with 1000 bootstrap replicates. Exon–intron structures were inferred by aligning genomic and cDNA sequences and visualized using the Gene Structure Display Server (GSDS 2.0; http://gsds.gao-lab.org/, accessed on 5 March 2025). Conserved motifs were identified with MEME Suite (v5.4.1; http://meme.nbcr.net/meme/intro.html, accessed on 5 March 2025) [42], setting the maximum number of motifs to 10 and motif width between 6 and 50 residues.

### 4.3. Chromosomal Localization, Duplication and Synteny

Chromosomal coordinates of *AsJAZ* genes were extracted from the garlic genome GFF3 file and visualized using TBtools v1.09876 (https://github.com/CJ-Chen/TBtools-II, accessed on 10 March 2025) [43]. Tandem and segmental duplication events within the garlic genome were analyzed using MCScanX (http://chibba.pgml.uga.edu/mcscan2/, accessed on 10 March 2025); however, no such duplication events were identified for the *AsJAZ* family. To further explore the evolutionary history of these genes, interspecific synteny among garlic, onion, and Welsh onion was investigated. The resulting collinear relationships were mapped and displayed using the “Dual Synteny Plotter” module in TBtools [43].

### 4.4. Cis-Regulatory Element Analysis, Protein–Protein Interaction Network and Gene Ontology Enrichment

Promoter regions (2000 bp upstream of the ATG) for each *AsJAZ* gene were extracted with TBtools and analyzed for cis-acting elements using PlantCARE (http://bioinformatics.psb.ugent.be/webtools/plantcare/html/, accessed on 15 March 2025) [44]. Identified elements were categorized by function—hormone response, light response, stress response and visualized as heatmaps in HemI v1.0.3.7. For functional annotation, the full-length protein sequences of AsJAZs were submitted to the EggNOG database (http://eggnog5.embl.de/, accessed on 15 March 2025) [45]. Gene Ontology (GO) annotation was then performed and visualized using WEGO (https://wego.genomics.cn/, accessed on 15 March 2025) [46]. The protein–protein interaction (PPI) network of the AsJAZ family was constructed using Cytoscape software (version 3.9.1). First, the protein sequences of *JAZ* genes were submitted to the STRING database (version 12.0, https://string-db.org/, accessed on 15 March 2025) for interaction prediction. The minimum required interaction score was set to a high-confidence threshold (≥0.700) to ensure the reliability of the predicted interactions. The resulting interaction data were then imported into Cytoscape for network visualization and analysis.

### 4.5. Plant Material and Stress Treatments

Garlic cultivar ‘Zipi’, maintained at the Onion and Garlic Germplasm Resource Nursery of Inner Mongolia Agricultural University, was used in this study. Uniform, disease-free garlic cloves were surface-sterilized in 70% ethanol for 5 min and then rinsed with sterile water. The plants were grown using half-strength Hoagland nutrient solution as the growth medium and germinated at 23–25 °C under a 16 h light/8 h dark cycle until seedlings reached 12–15 cm (≈3 weeks). For stress induction and expression analysis, seedlings were exposed to 39 °C (high-temperature stress) [47], 200 mM NaCl (salt stress) [48] or 100 µM MeJA (methyl jasmonate) [49]. To capture early transcriptional responses, leaf samples were harvested at 6, 12, and 24 h post-treatment, flash-frozen in liquid nitrogen, and stored at −80 °C. For each treatment, three independent biological replicates were collected.

### 4.6. Quantitative Real-Time PCR

Total RNA was extracted using the RNAprep Pure Plant Kit (Tiangen Biotech, Beijing, China) and reverse-transcribed with PrimeScript™ RT Master Mix (TaKaRa Biotechnology, Dalian, China). Gene-specific primers (Table S2) were designed with Primer 5 (Premier Biosoft, Palo Alto, CA, USA) and synthesized by Sangon Biotech (Shanghai, China). qRT–PCR was performed on an FTC-3000P Real-Time PCR System (Funglyn Biotech, Toronto, ON, Canada) using SYBR^®^ Premix Ex Taq™ II (Tli RNaseH Plus, RR820A; TaKaRa Biotechnology). *AsGAPDH* and *AsUBQ* were initially tested as candidate reference genes based on previous garlic studies, but due to their variable expression under heat (39 °C), salt (200 mM NaCl) and MeJA (100 µM) stresses, *Asβ-actin* was ultimately selected for normalization due to its stable expression. *Asβ-Actin* served as an internal control [50], and the control (CK) plants were used as an external control [51]. Relative expression levels were calculated by the 2^−ΔΔCT^ method [52]. Regarding tissue-specific expression analysis, the data were obtained from public transcriptome datasets available in the NCBI SRA database (PRJNA243415).

### 4.7. Subcellular Localization of AsJAZ Proteins

The coding sequences of *AsJAZ* genes (excluding stop codons) were cloned into the plant expression vector pCAMBIA1302, which contains a CaMV 35S promoter to drive constitutive expression in plants. The resulting GFP fusion constructs and empty vector (control) were introduced into *Agrobacterium tumefaciens* strain GV3101 using the heat-shock method [53]. Transformed *Agrobacterium* cultures were grown, harvested, and resuspended in infiltration buffer (10 mM MES, 10 mM MgCl_2_, 200 μM acetosyringone, pH 5.6) to an OD600 of 0.8. Agroinfiltration was performed by infiltrating the bacterial suspension into the abaxial sides of fully expanded leaves from 4 to 6-week-old *Nicotiana benthamiana* plants using a needleless syringe. For co-localization analysis, *Agrobacterium* cultures harboring GFP constructs were mixed at a 1:1 ratio with those containing organelle-specific markers (NLS-mCherry). After 48–72 h of incubation under standard growth conditions, fluorescence signals of GFP and mCherry were observed using a Nikon C2 Plus confocal laser-scanning microscope (Nikon, Tokyo, Japan). Bright-field and merged images were also captured. All experiments were performed in at least three independent biological replicates.

### 4.8. Yeast Two-Hybrid (Y2H) Assay

The physical interaction between AsJAZ17 and the MYC2 transcription factor (Asa7G03408.1) was verified using the Matchmaker^®^ Gold Yeast Two-Hybrid System (TaKaRa Biotechnology, Dalian, China). The full-length coding sequences of *AsJAZ17* and *Asa7G03408.1* were cloned into pGBKT7 (bait) and pGADT7 (prey) vectors, respectively, and co-transformed into Saccharomyces cerevisiae strain Y2HGold. Transformed yeast cells were initially grown on SD/−Trp/−Leu medium. For interaction assays, confirmed colonies were resuspended and adjusted to an OD_600_ of 0.1, followed by 10-fold serial dilutions. The yeast suspensions were spotted onto selective media, including SD/−Trp/−Leu/−His and SD/−Trp/−Leu/−His/−Ade, both supplemented with 30 mM 3-AT and X-α-Gal (40 μg/mL) to detect reporter gene expression. The plates were incubated at 30 °C for 3–5 days. Positive and negative controls were included as references.

### 4.9. Arabidopsis Transformation and Generation of Transgenic Lines

To generate the overexpression construct, the full-length coding sequence (CDS) of *AsJAZ17* was amplified and cloned into the pBI121 vector under the control of the constitutive cauliflower mosaic virus 35S (CaMV 35S) promoter. The recombinant plasmid pBI121-AsJAZ17 was subsequently transformed into *Agrobacterium tumefaciens* strain GV3101. The overexpression construct was introduced into *Arabidopsis thaliana* (ecotype Columbia-0, Col-0) via the Agrobacterium-mediated floral dip method. Putative transgenic plants were screened on half-strength Murashige and Skoog (1/2 MS) medium supplemented with 50 mg/L kanamycin. Through self-crossing and antibiotic resistance analysis, three independent T3 homozygous overexpression lines (designated as OE#3, OE#4, and OE#7) were identified and utilized for subsequent experiments.

### 4.10. Salt Stress Treatment and Physiological Measurements

For salt tolerance assays, surface-sterilized seeds of the wild-type (WT) and transgenic lines were sown in a substrate mixture (vermiculite:perlite:peat moss = 1:1:1, v/v/v) and cultivated under standard conditions (22 °C, 16 h light/8 h dark) for 30 days. The plants were then subjected to salt stress by irrigation with a 100 mM NaCl solution. This specific concentration was selected to effectively differentiate physiological and phenotypic responses in the salt-sensitive *Arabidopsis* background without causing premature seedling mortality. To allow sufficient time for stress-induced phenotypic and physiological differences to manifest, observations were recorded, and samples were collected 72 h post-treatment. Specifically, 0.2 g of leaf tissue was harvested, flash-frozen in liquid nitrogen, and ground into a fine powder. The absorbance for the subsequent enzymatic and non-enzymatic assays was recorded using a UH5300 UV-Vis spectrophotometer (Hitachi, Tokyo, Japan). SOD activity was determined using the nitroblue tetrazolium (NBT) photochemical reduction method [54]. POD activity was measured via the guaiacol method [55], and CAT activity was assessed using a colorimetric assay [56]. The MDA content was determined using the thiobarbituric acid (TBA) reaction method to evaluate lipid peroxidation [57]. To assess the accumulation of ROS, the H_2_O_2_ concentration was measured via the titanium sulfate colorimetric assay [58], and the production rate of the O_2_^−^ was estimated by the hydroxylamine oxidation method [59].

### 4.11. Statistical Analysis

All experimental data were obtained from at least three independent biological replicates and are presented as the mean ± standard deviation (SD). Statistical significance was evaluated using one-way analysis of variance (ANOVA) followed by Duncan’s multiple range test or Student’s t-test to determine differences between groups. A *p*-value < 0.05 was defined as the threshold for statistical significance. All statistical analyses and figure construction were performed using SPSS 22.0 (IBM, Armonk, NY, USA) and Origin 2021 (OriginLab Corporation, Northampton, MA, USA).

## 5. Conclusions 

In summary, this study identified 28 *AsJAZ* genes in garlic, revealing a significant lineage-specific expansion. While the family maintains core conserved domains, the spatial divergence between the strictly nuclear AsJAZ17 and the nucleocytoplasmic AsJAZ16 points to a sophisticated level of functional compartmentalization. Integrated analysis using PPI modeling and Y2H assays confirmed that AsJAZ17 physically interacts with MYC2, establishing it as a key regulator of salt stress responses within the conserved COI1-JAZ-MYC2 module. Functional validation demonstrated that *AsJAZ17* overexpression significantly enhances salt tolerance by optimizing the growth-defense trade-off and reinforcing the ROS-scavenging system. These findings provide a comprehensive understanding of the garlic *JAZ* family and identify *AsJAZ17* as a promising candidate for improving stress resilience in *Allium* crops through molecular breeding.

## Figures and Tables

**Table 1:** The characteristics of the *AsJAZ* family.

No.	Name	Sequence ID	Number of Amino Acid	Molecular Weight	Theoretical pI	Instability Index	Aliphatic Index	Grand Average of Hydropathicity	Subcellular Localization
1	AsJAZ1	Asa0G00889.1	207	22,316.56	9.5	52.66	89.61	−0.155	Cytoplasm
2	AsJAZ2	Asa0G01028.1	170	18,171.63	7.77	47.06	73.41	−0.321	Nucleus
3	AsJAZ3	Asa0G01029.1	110	12,400.18	8.96	64.27	78.09	−0.44	Nucleus
4	AsJAZ4	Asa0G02987.1	136	15,408.72	9.45	52.07	86.18	−0.271	Nucleus
5	AsJAZ5	Asa0G05575.1	168	19,031.67	8.85	53.25	77.8	−0.524	Nucleus
6	AsJAZ6	Asa0G05603.1	149	16,788.39	9.49	59	78.59	−0.483	Nucleus
7	AsJAZ7	Asa1G00005.1	152	17,308.96	9.37	46.67	82.11	−0.45	Nucleus
8	AsJAZ8	Asa1G00028.1	149	16,936.64	9.44	58.79	77.92	−0.441	Nucleus
9	AsJAZ9	Asa1G00030.1	153	17,168.61	9.76	51.07	76.67	−0.568	Nucleus
10	AsJAZ10	Asa1G00038.1	147	16,348.55	9.38	68.11	82.38	−0.564	Nucleus
11	AsJAZ11	Asa1G00039.1	165	18,475.86	7.7	52.85	79.88	−0.552	Nucleus
12	AsJAZ12	Asa1G00040.1	158	17,504.78	9.12	59.1	81.58	−0.47	Cytoplasm
13	AsJAZ13	Asa1G00041.1	157	17,720.52	9.66	48.35	82.04	−0.441	Nucleus
14	AsJAZ14	Asa1G00043.1	163	18,230.58	8.5	51.61	76.63	−0.547	Cytoplasm
15	AsJAZ15	Asa1G00044.1	163	18,210.59	8.5	53.31	77.24	−0.536	Cytoplasm
16	AsJAZ16	Asa1G01572.1	174	18,939.62	8.71	53.04	83.1	−0.364	Cytoplasm
17	AsJAZ17	Asa2G00577.1	211	22,852.19	9.4	36.56	75.97	−0.434	Nucleus
18	AsJAZ18	Asa3G00235.1	131	15,283.6	9.78	69.77	59.62	−0.944	Nucleus
19	AsJAZ19	Asa3G00571.1	349	39,802.68	8.78	44.66	87.45	−0.349	Nucleus
20	AsJAZ20	Asa3G00573.1	287	33,162.1	4.94	47.2	71.01	−0.984	Cytoplasm
21	AsJAZ21	Asa3G00575.1	171	19,314.05	8.5	51.21	82.16	−0.437	Nucleus
22	AsJAZ22	Asa3G04941.1	217	25,261.11	9.82	62.33	66.08	−0.722	Nucleus
23	AsJAZ23	Asa3G05094.1	170	19,239.87	8.5	53.81	74.06	−0.546	Nucleus
24	AsJAZ24	Asa4G00559.1	224	24,690.07	9.17	58.3	74.82	−0.535	Nucleus
25	AsJAZ25	Asa4G01523.1	156	17,173.55	9.62	41.9	86.99	−0.216	Cytoplasm
26	AsJAZ26	Asa4G01696.1	180	19,532.2	9.71	38.32	69.94	−0.547	Cytoplasm
27	AsJAZ27	Asa5G03418.1	107	12,394.54	10.41	39.76	89.35	−0.328	Nucleus
28	AsJAZ28	Asa7G05781.1	172	19,288.17	9.57	40.19	78.31	−0.509	Nucleus

## Data Availability

The original contributions presented in this study are included in the article/Supplementary Material. Further inquiries can be directed to the corresponding author.

## Supplementary Materials

The following supporting information can be downloaded at: https://www.mdpi.com/article/10.3390/plants15101543/s1, Table S1: List of Conserved Motifs and Corresponding Sequences in AsJAZs. Table S2: List of the primers used in the study. Figure S1. A Predicted Protein–Protein Interaction (PPI) Network of the *A. sativum* JAZ Family and Key Interactors.
