# Supplementary material for: Genome-Wide Identification of the JAZ Gene Family in Garlic (Allium sativum L.) and the Functional Role of AsJAZ17 in Salt Tolerance
Source: Plants (Basel). 2026 May 19;15(10):1543. doi: 10.3390/plants15101543 (PMC13210613; doi:10.3390/plants15101543)
Supplement: Supplementary file 1 [file plants-15-01543-s001.zip › Supplementary Materials Figure S1.pdf]

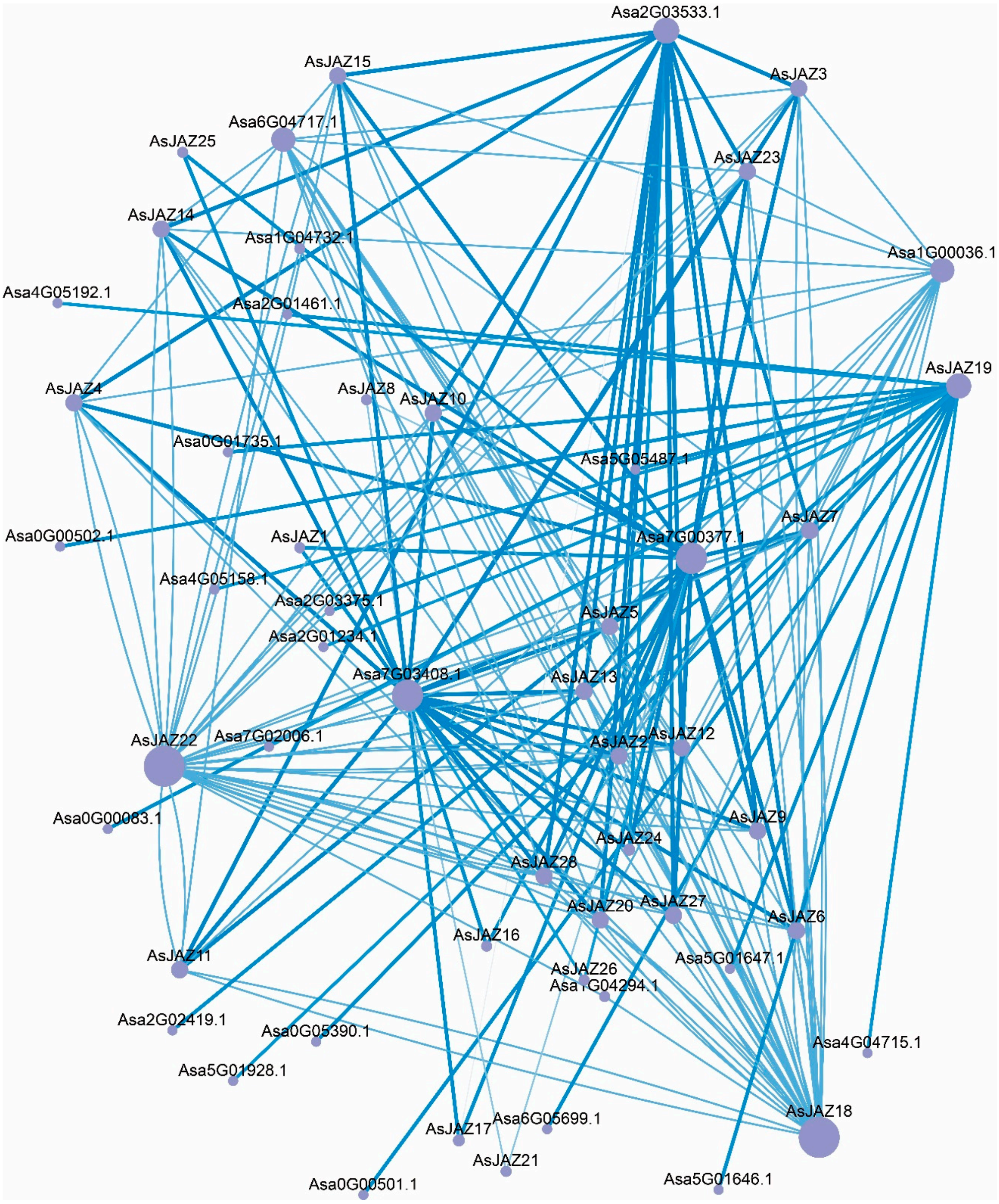

Figure S1. A Predicted Protein–Protein Interaction (PPI) Network of the *A. sativum* JAZ Family and Key Interactors. A detailed interaction network based on predictive PPI modeling, complementary to the broader functional overview in Figure 8. This visualization details the intricate web of both internal family-level connections and external associations within and surrounding the *AsJAZ* family. Key hubs such as AsJAZ18 and AsJAZ22 are positioned as essential mediators connecting distinct JAZ branches and other core transcription factors, reinforcing the role of JAZ proteins as multi-faceted signaling nexus points within larger protein complexes.
